# 地中海饮食对肺癌相关性影响的研究进展

**DOI:** 10.3779/j.issn.1009-3419.2024.106.09

**Published:** 2024-04-20

**Authors:** Minglang GAO, Kai LAI, Zilong LU, Yi LIU, Ning LI, Qing GENG

**Affiliations:** 430060 武汉，武汉大学人民医院胸外科; Department of Thoracic Surgery, Renmin Hospital of Wuhan University, Wuhan 430060, China

**Keywords:** 肺肿瘤, 地中海饮食, 饮食, 健康, 蔬菜, 水果, Lung neoplasms, Mediterranean diet, Diet, Health, Vegetables, Fruits

## Abstract

肺癌是当今世界十大死因之一，它以其高死亡率令全世界深感忧愁。在现代医学研究中，研究人员正致力于深入挖掘肺癌发生和发展的不同影响因素，以降低其发病率，提高肺癌的治疗成功率，改善肺癌患者的预后。地中海饮食（Mediterranean diet, MD）是一种特殊的膳食结构，它以吃蔬菜、水果、粗粮、豆类和低脂鱼类为主，被报道具有抗炎、抗氧化、降脂等作用。近年来的研究发现，MD可能会在一定程度上预防肺癌，并对其发病和进展产生重要影响。本文旨在对相关文献进行综述，总结及分析性地探讨MD对肺癌发病及进展的影响，从而更好地为预防和治疗肺癌提供研究方向。

## Abstract (en)

Lung cancer is one of the top 10 causes of death in the world today, and it is a great concern worldwide for its high mortality rate. Currently, the researchers are digging into various factors influencing the occurrence and development of lung cancer in order to increase the odds for curing lung cancer, improve the prognosis of lung cancer patients as well as reduce its morbidity. The Mediterranean diet (MD) is a special dietary structure that is based on eating vegetables, fruits, coarse grains, legumes and low-fat fish, which have anti-inflammatory, antioxidant and lipid-lowering effects. Recent studies have revealed that the MD may prevent lung cancer occurrence to some extent and inhibit its development. The purpose of this paper is to summarize and analytically discuss the effects of the MD on the oncogenesis and development of lung cancer through a review of the relevant literatures, thus to provide references for MD to prevent and treat lung cancer.

癌症是全世界主要的死亡原因之一，作为一个重大的全球健康问题，每年造成全球数百万人死亡^[1]^。研究数据^[2]^表明肺癌在全球癌症相关死亡率中高居首位，是许多医学工作者研究的临床难点。肺癌的发病机制是一个非常复杂的过程，涉及到多种因素，最常见的个体危险因素是吸烟，长期吸烟的人群比不吸烟的人群更有可能患上肺癌^[3]^。尽管吸烟是主要的危险因素，被认为在癌症死亡原因中占22%，但其他因素，包括长期不良的饮食习惯、长期不运动以及肥胖等个体的遗传特征，都会造成正常肺组织细胞进展为肿瘤细胞，在一定程度上加速恶性肿瘤的癌前病变^[4,5]^。目前免疫治疗和靶向治疗的重大突破，显著提高了肺癌患者的预后，延长了生存时间，但是免疫治疗和靶向治疗耐药性也屡见不鲜^[6]^。多项流行病学研究^[7,8]^结果表明，适当食用蔬菜和水果可以预防癌症并在一定程度上降低癌症发生率，改变饮食习惯甚至有助于避免30%-50%的癌症发病。

地中海饮食（Mediterranean diet, MD）最开始由Keys等^[9]^于20世纪80年代提出，现如今被认为是世界上最健康的饮食模式之一。其潜在的健康益处已在大量文献^[10,11]^中得到广泛报道，其特点是大量食用蔬菜和水果、豆类、海鲜、坚果和全谷物，适量食用奶制品、红肉和葡萄酒，以及少量食用加工食品和含糖饮料。MD的摄入有助于提高健康水平，且可降低肥胖、代谢综合征、高血压和冠状动脉疾病等慢性疾病的发病率^[12]^。同样，研究^[13⇓⇓⇓⇓⇓-19]^显示MD可降低不同类型癌症（如肺癌、结肠癌、胃癌、乳腺癌、直肠癌、食道癌和口腔癌等）的发病率，表明其对一些恶性肿瘤包括肺癌有一定的预防作用，但是其对肺癌的影响尚未有深入的研究，因此，本文就MD对肺癌的作用进行分析，并总结MD的不同组分在其中的主要作用机制。

## 1 MD与肺癌的相关临床研究

根据世界卫生组织的数据，2020年全球新诊断的肺癌病例达到221万例，而报告的肺癌死亡病例为180万例。据估计，到2035年肺癌死亡人数将增加到300万^[20]^。国内多数研究以风险因素或流行病学调查为基础，探讨MD和肺癌的联系，Du等^[21]^的meta分析结果表明，MD模式与肺癌风险之间存在显著的负相关[风险比（relative risk, RR）为0.82，95%CI：0.74-0.92]，异质性较高（I^ 2^=59.9%, P<0.05），基于最低MD评分（MD score, MDS）（0分），每增加3分，肺癌风险就会降低9%，从而表明MD模式是肺癌的保护因素。该研究作为首个探讨MD模式与一般人群患肺癌风险之间关系的研究，其结果对于降低肺癌的发病率具有重要意义。

国外研究进展也表明，MD在肺癌预防和治疗方面具有重要的作用，Bahrami等学者^[22]^通过对8项队列研究和1项病例对照进行系统评价和meta分析所得出的结论表明，长期保持MD可以使得肺癌的患病风险降低16%（RR=0.84，95%CI: 0.77-0.91；I^2 ^=52%，异质性P=0.03），且MDS每提高2分，肺癌风险就会降低6%，这与Du等^[21]^的研究结果类似。另外，在具有既往吸烟史的研究对象亚组中观察到MD依从性与肺癌的患病风险之间呈负相关，即表明戒烟的人通过遵循健康的生活方式可能会变得更加健康，包括更好的饮食和更多的身体活动，这可能对整体健康和癌症风险产生协同效应。当然这也需要进一步调查来探索研究对象戒烟后的整体健康行为，以证实这项研究的结果。

Hawrysz等^[23]^纳入了2013至2017年439名年龄在45-80岁的波兰成年男性吸烟者，此项病例对照学研究探讨了坚持MD与患肺癌风险之间的关系，结果显示，坚持MD使中度吸烟者中肺癌风险降低41%，低度吸烟者中肺癌风险降低66%，但在重度吸烟者中，MD与肺癌风险没有显著的相关性。Hodge等^[24]^对35,303例墨尔本当地居民进行了18年随访，总共发现了403例肺癌病例，前瞻性地研究膳食炎症指数（dietary inflammatory index, DII）和MDS与肺癌之间的关系，结果显示，MDS与肺癌风险呈负相关，尤其是吸烟者，高DII即更容易引发炎症的饮食，与肺癌风险呈正相关，说明对吸烟者来说，MD在一定程度上可以降低患肺癌的风险。Gioxari等^[25]^的另一项前瞻性随机对照试验，对实施MD 3个月后肺癌患者的炎症史和营养状况进行评估，共纳入30例肺癌患者，对照组接受一般营养指南，MD组提供个性化的MD营养计划，研究结果显示对照组患者的晚期肺癌炎症指数（advanced lung cancer inflammation index, ALI）显著高于MD组患者（P=0.003），说明MD有望改善晚期肺癌患者的预后。

## 2 机制分析

### 2.1 蔬菜和水果

MD中的蔬菜和水果含有大量的营养成分，如维生素、矿物质、吲哚类物质和黄酮类物质等，可以通过调节特定的细胞信号通路进而抑制肿瘤细胞增殖、诱导凋亡，降低肺癌发病率^[26,27]^。其中的维生素A、C和E已被证明具有预防肺癌发生的作用，Yong等^[28]^在一项营养调查流行病学研究队列中发现维生素A、C和E的摄入量与肺癌发病率有关，结果证明了饮食中的维生素E和C以及类胡萝卜素对肺癌发病具有抑制作用，但其效果会因吸烟的强度而改变。同样的，Alberg等^[29]^的研究报告也显示吸烟会影响血液中维生素C的水平，血液循环中的维生素C浓度随着每天吸烟数量的增加而降低。维生素D是人体内一种重要的激素，其有多种生物学作用^[30]^，同时也被认为具有抗肿瘤作用，体外研究^[31,32]^结果表明，维生素D可降低小细胞肺癌细胞的增殖能力并诱导细胞凋亡，Nakagawa等^[33]^的动物实验则证明了维生素D可以抑制肺癌的转移。另外有学者发现，一些蔬菜例如大蒜和洋葱中的硒化物能促进环腺甘酸的积累和增强人体免疫反应，进而抑制肿瘤细胞分裂与生长^[34,35]^，茄子中所含有的一些成分如龙葵碱，对肺癌和一些消化道肿瘤有一定的抑制作用^[36]^，而一些海产品包括海带、紫菜等中所含有的不饱和脂肪酸和β-葡聚糖等都能抑制肿瘤细胞的生长^[37]^。

吲哚-3-甲醇（indole-3-carbinol, I3C）是一种存在于卷心菜和甘蓝等蔬菜中的植物化学物质，通常认为其具有防癌和抗癌的作用。Kassie等^[38]^的研究表明I3C在肺癌发生发展中的抑制作用部分是通过诱导细胞凋亡和抑制细胞增殖来实现的。Choi等^[39]^研究表明，I3C能抑制肺癌细胞的分裂周期并在A549细胞系上观察到了结果，进一步证明I3C通过提高细胞周期蛋白依赖性激酶抑制剂以及P53的表达水平来阻止细胞分裂周期，并通过激活Fas和胱氨酸-8级联途径诱导细胞凋亡，这是关于I3C对肺癌细胞凋亡作用分子机制的首次报道。Xiao等^[40]^调查了I3C在肺癌发展中的放疗敏感性和化学预防作用，结果显示，用I3C预处理表皮生长因子受体（epidermal growth factor receptor, EGFR）阳性的人肺腺癌细胞，可明显增强其对γ射线的辐射敏感性。Qian等^[41]^的研究利用了烟草诱导的小鼠肺癌模型评估I3C对肺腺癌的化学预防作用，结果显示I3C通过受体酪氨酸激酶/PI3K/Akt信号通路抑制肺腺癌进展。总之，I3C可以通过影响参与细胞增殖和凋亡通路来抑制肺癌细胞的生长，提高EGFR阳性肺癌细胞的EGFR蛋白表达水平来促进肺癌细胞的放疗敏感性，并通过受体酪氨酸激酶/PI3K/Akt信号通路对肺腺癌发挥化学预防作用。

黄酮类化合物是蔬菜水果中的一类重要植物化学物质，研究^[42,43]^表明，经常食用含有类黄酮的MD可以减少大多数恶性肿瘤的发病率，如肺癌、结肠癌和乳腺癌等。Chian等^[44]^的研究证明，黄酮类化合物可以通过激活核因子E2相关性因子2（nuclear factor E2 related factor 2, Nrf2）通路在体内或体外抑制肺癌细胞的增殖，从而抑制肿瘤生长，表明黄酮类化合物可以作为非小细胞肺癌化疗的辅助剂。Chen和Ruan等的研究^[45,46]^则表明黄酮类化合物可以通过抑制E-Cadherin蛋白抑制上皮间充质转化，降低肿瘤细胞的迁移能力。还有研究^[47]^表明，肺癌细胞经黄酮类化合物处理后，以浓度依赖的方式降低其中肿瘤相关巨噬细胞受体酪氨酸激酶（receptor tyrosine kinases, RTKs）的表达水平，证明RTKs可能是黄酮类化合物抑制肺癌增殖的靶点。

### 2.2 肉类和红酒

尽管既往研究^[48]^表明食用猪肉和鸡肉对肺癌具有抑制作用，但大多数报告^[49,50]^称食用大量的肉类会增加肺癌的患病风险，因为大部分肉类都含有一些脂肪类物质，包括胆固醇等，该类物质可使细胞膜通透性发生改变，从而有利于致癌物的进入。众所周知，烹饪等加热食物的方式会导致氨基酸分解产生杂环芳香胺，而杂环芳香胺具有高度致癌性，可以诱发多种不同的肿瘤^[51]^。烹饪的方式以及火候的把控都会产生影响，因为肉类在高温下特别是在油炸时，会形成杂环胺，而在烤肉或烧烤时，会形成多环芳香烃（polycyclic aromatic hydrocarbonspahs, PAHs），多环芳香烃最终将导致DNA突变并增加癌症发病风险^[52]^。

对于观察到的猪肉和鸡肉可以降低癌症的发病率，一个合理的解释是由于这些食物经常与蔬菜水果一起吃，所以造成这些抑制作用更可能是取决于蔬菜和水果当中的物质，另外，鸡肉的脂肪含量比其他类型的肉要低一些^[53]^。另一个合理的解释是，在地中海沿岸，肉类（尤其是猪肉和牛肉）在食用时往往会搭配红酒和葡萄酒，这些酒类中含有白藜芦醇^[54]^和单宁酸，有研究^[55]^显示，红酒对肺癌具有一定的抑制作用。De Stefani等^[56]^进行的一项病例对照研究中也发现了同样的影响，证实食用肉类和饮用葡萄酒之间的这种相关性。

有关鱼类于肺癌相关性的研究并不多，日本研究者Takezaki等^[57]^于2001年招募了607例腺癌患者和4153例对照人员进行了病例对照研究，结果表明，食用煮熟的鱼或者生鱼可以降低日本人患肺癌的风险，特别是在食用频率超过每周一次的情况下。多不饱和脂肪酸（polyunsaturated fatty acids, PUFAs）和环氧化酶-2（cyclooxygenase-2, COX-2）在肺癌发展中的作用已进行大量研究，而PUFAs广泛存在于鱼类制品（例如干鱼或盐渍鱼和新鲜鱼片等）中，鱼油中的二十碳五烯酸（eicosapentaenoic acid, EPA）和二十二碳六烯酸（docosahexaenoic acid, DHA）也会对肺癌的发生发展产生抑制作用^[57]^。挪威的一项研究^[58]^报告了鱼肝油补充剂与肺癌风险之间呈现负相关，原因是PUFAs的高摄入可以降低肺癌的发病率。然而，鱼油如果长期暴露于空气、阳光照射或者高温环境中，会由于氧化作用和其他化学效应而迅速降解，因此PUFA浓度也会随着烹饪而下降^[57]^。COX-2在体内作为一种重要的酶参与花生四烯酸等物质的代谢过程，研究^[59]^发现其不仅参与炎症反应，而且还参与致癌作用，PUFAs通过与COX-2不同的机制来抑制花生四烯酸的代谢，从而调节致癌过程^[60]^。

### 2.3 橄榄油和谷物

橄榄油作为MD模式中的主要脂肪来源，主要富含包括单不饱和脂肪酸、三萜和多酚，如酚醇（如羟基酪醇）、环烯醚萜类（如橄榄苦苷和油橄榄素）、木脂素（如松脂醇）等物质^[61]^，其作用至关重要。有研究^[62]^表明，橄榄油对预防癌症具有一定的保护作用，例如橄榄油的酚类和脂质部分可以防止结直肠癌的发生，Gill等^[62]^发现橄榄油中的苯酚提取物通过阻碍整合素与各种类型的细胞外基质蛋白的结合来防止体外细胞附着，整合素对于细胞侵袭和迁移过程至关重要，是癌发生起始、促进和转移的基本步骤，这也首次证明了橄榄油中酚类物质的抗侵袭作用。

油酸作为初榨橄榄油中的主要脂肪酸，占比高达70%-80%，其抗氧化特性有助于通过减少促炎分子的形成来保护细胞不被氧化，油酸下调COX2的表达，这在结直肠癌的发展中起着重要作用^[63]^。此外，油酸还诱导HT-29细胞凋亡，下调20%乳腺癌中的Her-2癌基因表达，抑制癌基因Her-2在多种人类癌症的病因、侵袭性进展和转移中发挥着关键作用^[64]^。橄榄油中角鲨烯含量虽然含量不高，但动物研究^[63]^表明，局部角鲨烯对化学诱发的皮肤癌具有抑制作用，可以降低皮肤癌发病率。木脂素作为橄榄油中不可或缺的组分，具有抗氧化活性和抗病毒活性，可以抑制皮肤肿瘤、乳腺癌、结肠癌、肺癌等多种肿瘤的发生发展^[65]^。

谷物通常被定义为包括面包、谷类食品和面食等主粮食品，作为MD中的主食，在这一饮食模式中起着不可或缺的作用^[66]^。国外的一项针对全谷物和精制谷物的meta分析的结果显示，全谷物摄入量可以使癌症风险降低6%-12%^[67]^。谷物是膳食纤维的重要来源，而膳食纤维已被证明与癌症的风险降低有关，纤维素有助于维持肠道健康，促进正常的消化和排便，同时可能减少有害物质在肠道内停留的时间，同时，膳食纤维也可在肠道中发酵，产生有益的短链脂肪酸，有助于维持肠道菌群的平衡，这与免疫系统的正常功能和癌症抵抗力相关^[68]^。谷物含有丰富的抗氧化物质，如维生素E、硒等，这些成分有助于中和自由基，减少细胞氧化应激，从而降低癌症的风险^[69]^。另外，MD中的谷物主要是整粒谷物，如全麦、糙米等，它们具有较低的升糖指数，通过维持较稳定的血糖水平，谷物有助于预防胰岛素抵抗和糖尿病^[70]^，从而减少与这些疾病相关的癌症风险。这些研究结果表明，饮食中的多种化合物对癌细胞的发生发展有重要的影响，而相较于正常饮食，进行MD饮食的患者由于摄入多量的组分可能会对癌细胞产生抑制生长的作用，同时对癌前病变也有预防作用，综合以上结果也说明，MD作为一种饮食结构，其具有的多种组分共同对肺癌发生发展产生影响而非单独成分。然而，尚未有直接的证据表明MD饮食对肺癌的直接作用。

## 3 总结

近年来，肺癌已成为全球范围内发病率和死亡率最高的恶性肿瘤，目前我们对肺癌发生、发展、转移和耐药的分子机制的认识还很有限。已确定的风险因素包括遗传、环境和生活方式等，因此，我们需要探讨肺癌的保护因素，以降低肺癌的发病率，提高肺癌的防治水平。

在文中，我们已经探讨了几种可能的机制将MD模式与肺癌发病率的降低联系起来，营养素和食物通过复杂的组合构成日常饮食，维生素C、类胡萝卜素等单一营养素在预防慢性疾病的过程中发挥了巨大作用，但我们仍然需要探索不同饮食结构的组成来提高患者的生活质量^[71]^。蔬菜、水果、全谷物、鱼类和大豆制品等都被证明可以降低肺癌的发病风险^[72]^。富含这些食物成分且高脂乳制品和红肉含量较低（MD模式）的饮食一直被认为是健康饮食，研究^[73,74]^表明长期保持MD的人群患心血管疾病、糖尿病和癌症等慢性非传染病的概率大幅降低。MD是膳食纤维、维生素、矿物质和多酚等抗癌剂的良好来源，具有很强的抗氧化能力，其对肺癌的抑制作用可以通过其预防DNA损伤、氧化应激和炎症的能力来解释。肠道内的微生物群可以将食物中的膳食纤维发酵成短链脂肪酸，其已被证明有益于宿主免疫力和包括肺在内的器官的新陈代谢^[68]^。此外，动物研究^[75]^还发现膳食纤维可以通过改变肺部微生物群的组成来重塑免疫环境。MD中常见的鱼类制品富含PUFAs脂肪酸，有助于阻止肿瘤细胞增殖、存活、血管生成和炎症反应。橄榄油作为MD中几乎唯一的食用油，其含有的油橄榄醛可以通过靶向c-MET和COX-2抑制肺癌的进展和转移^[76]^。MD中经常饮用的红酒里含有的多酚可以抑制DNA聚合酶III基因并促进T辅助细胞以及抑制DNA甲基转移酶和组蛋白脱乙酰酶，这些都可能会减缓肿瘤细胞的发生和发展^[77]^。此外，MD中还含有丰富的维生素A和β-胡萝卜素、黄酮类化合物、硒和维生素 E等物质，这些都可以降低肺癌的发生率^[78]^。然而，地中海饮食作为一种饮食模式，包含了多种成分，为了明确地了解它对肺癌的影响，可能需要研究这些成分的组合效应，而不仅仅是单个成分的影响。

未来还需开展针对肺癌患者指导饮食干预等项目，以进一步评估MD对疾病的影响以及探究MD是否能够改变患者饮食习惯和降低肺癌的复发率。总的来说，本文总结了近年来MD与肺癌发生发展相关的研究进展，对MD模式中含有的部分食物组分进行了单独分析，并阐述了可能的影响机制。通过上述内容，我们可以看出，MD对肺癌相关性影响是明显的，有助于降低疾病发生率，目前尚无法确定MD在肺癌预防与治疗中的优势究竟是哪个方面更为明显。MD越来越被认为是健康饮食的典范，并一直与许多健康益处相关联。然而，仍然需要大规模、长期的随机临床试验和随访研究来充分评估这种饮食的潜在作用并获得更为科学准确的结论，这将为肺癌发病的预防和肺癌患者的治疗提供有用的参考信息。
